# Root-associated fungal communities in three Pyroleae species and their mycobiont sharing with surrounding trees in subalpine coniferous forests on Mount Fuji, Japan

**DOI:** 10.1007/s00572-017-0788-6

**Published:** 2017-07-13

**Authors:** Shuzheng Jia, Takashi Nakano, Masahira Hattori, Kazuhide Nara

**Affiliations:** 1 Department of Natural Environmental Studies, Graduate School of Frontier Sciences, The University of Tokyo, 5-1-5 Kashiwanoha, Kashiwa, Chiba 277-8563 Japan; 2 Mount Fuji Research Institute, Fujiyoshida, Yamanashi, Japan; 3 Laboratory of Metagenomics, Graduate School of Frontier Sciences, The University of Tokyo, Kashiwa, Chiba Japan

**Keywords:** Pyroleae, Mixotrophy, Mycorrhizal network, Niche overlap, ITS barcoding

## Abstract

Pyroleae species are perennial understory shrubs, many of which are partial mycoheterotrophs. Most fungi colonizing Pyroleae roots are ectomycorrhizal (ECM) and share common mycobionts with their Pyroleae hosts. However, such mycobiont sharing has neither been examined in depth before nor has the interspecific variation in sharing among Pyroleae species. Here, we examined root-associated fungal communities in three co-existing Pyroleae species, including *Pyrola alpina*, *Pyrola incarnata*, and *Orthilia secunda*, with reference to co-existing ECM fungi on the surrounding trees in the same soil blocks in subalpine coniferous forests. We identified 42, 75, and 18 fungal molecular operational taxonomic units in *P*. *alpina*, *P*. *incarnata*, and *O*. *secunda* roots, respectively. Mycobiont sharing with surrounding trees, which was defined as the occurrence of the same mycobiont between Pyroleae and surrounding trees in each soil block, was most frequent among *P*. *incarnata* (31 of 44 plants). In *P*. *alpina*, sharing was confirmed in 12 of 37 plants, and the fungal community was similar to that of *P*. *incarnata*. Mycobiont sharing was least common in *O*. *secunda*, found in only 5 of 32 plants. Root-associated fungi of *O*. *secunda* were dominated by *Wilcoxina* species, which were absent from the surrounding ECM roots in the same soil blocks. These results indicate that mycobiont sharing with surrounding trees does not equally occur among Pyroleae plants, some of which may develop independent mycorrhizal associations with ECM fungi, as suggested in *O*. *secunda* at our research sites.

**Electronic supplementary material:**

The online version of this article (doi:10.1007/s00572-017-0788-6) contains supplementary material, which is available to authorized users.

## Introduction

Most land plants perform photosynthesis and can live autotrophically. In contrast, however, some plants have evolved to obtain carbon from root-colonizing fungi (Leake 2004; Bidartondo 2005). Such carbon dependence on fungi is called mycoheterotrophy (MH) and ranges from full MH among achlorophyllous plants to partial MH (or mixotrophy), in which plants use both photosynthetic and fungus-derived carbon (Selosse and Roy 2009; Merckx 2013). In some plant species, the trophic mode changes with developmental stage. For example, most orchid species exhibit full MH during germination and the early stages of development (initial mycoheterotrophy) but become autotrophic as they mature (Warcup and Talbot 1967; Warcup 1973; Hynson et al. 2013a). However, at least 514 angiosperm species and 1 liverwort species are full MHs throughout their entire life cycle (Merckx 2013). Such a full MH lifestyle has multiple evolutionary origins and is evident in at least 46 independent plant lineages (Merckx 2013).

Mature partial MH plants may be more diverse than full MH plants (Selosse and Roy 2009; Merckx 2013). However, the exact diversity of partial MH plants is unknown and understudied (Selosse et al. 2016), partly because we cannot easily distinguish them from autotrophic plants by appearance, as both have green leaves. Analysis of ^13^C natural abundance ratios (δ^13^C) is potentially an effective approach for discriminating partial MH plants because fungi consistently have higher δ^13^C than autotrophic plants do. Enriched δ^13^C in some plants compared with surrounding autotrophic ones could potentially indicate the acquisition of fungal carbon and their partial MH status. Using this approach, some species of Pyroleae (syn. Pyroloideae, Angiosperm Phylogeny Website ver. 13, Selosse et al. 2016) have been suggested to be partial MH (Tedersoo et al. 2007; Zimmer et al. 2007; Hynson and Bruns 2009; Matsuda et al. 2012). Green orchids have also been referred to as partial MH species using this approach (Gebauer and Meyer 2003; Julou et al. 2005; Motomura et al. 2008; Roy et al. 2009). However, δ^13^C levels in plants are also affected by other factors, including water stress, light conditions, and stomatal traits (Farquhar et al. 1989), and enriched δ^13^C values alone are not sufficient to confirm partial MH status in some cases. Moreover, δ^13^C enrichment in green Pyroleae is inconsistent among species, conspecific populations, or conspecific individuals within a population (Tedersoo et al. 2007; Zimmer et al. 2007; Hynson and Bruns 2009; Hynson et al. 2015; Johansson et al. 2015). Thus, additional methods of evaluation are necessary to identify species as partial MH.

Additional evidence of partial MH plants is the identity of root-colonizing fungi. In forest ecosystems, many full MH orchids and Monotropoideae (an achlorophyllous subfamily in Ericaceae) species are colonized by ectomycorrhizal (ECM) fungi, while their autotrophic ancestors are associated with other fungal groups (Leake 2004; Bidartondo 2005; Selosse and Roy 2009; Strullu-Derrien et al. 2016). ECM fungi are ubiquitous in forest soil and receive abundant photosynthetic products from host trees (Hobbie 2006; Smith and Read 2008). By establishing common mycorrhizal networks (physical connection through shared ECM fungal mycelia) with surrounding trees, full MH plants can gain access to the abundant photosynthates of trees (Leake 2004; Bidartondo 2005; Selosse and Roy 2009; Motomura et al. 2010; Ogura-Tsujita et al. 2012). Such carbon transfer from trees to full MH plants has been demonstrated in ^14^C tracer experiments (Bjorkman 1960; McKendrick et al. 2000; Bidartondo et al. 2003). Green-leaved plants that are phylogenetically close to full MH and are associated with ECM fungi are often assumed to obtain carbon through common mycorrhizal networks in a similar way (Selosse et al. 2016).

A diverse set of root-colonizing ECM fungi, including *Cortinarius*, *Tricholoma*, *Russula*, *Thelephora/Tomentella*, *Sebacina*, *Wilcoxina*, and *Inocybe*, have been shown to colonize Pyroleae (Tedersoo et al. 2007; Zimmer et al. 2007; Massicotte et al. 2008; Vincenot et al. 2008; Hynson and Bruns 2009; Toftegaard et al. 2010; Hashimoto et al. 2012; Matsuda et al. 2012), which are phylogenetically rather close to full MH Monotropoideae (Lallemand et al. 2016). Because these ECM fungi are usually hosted by surrounding trees, it has been hypothesized that Pyroleae and surrounding trees share common mycorrhizal networks. However, this hypothesis has not been tested rigorously, as most of the previous studies did not examine the ECM tips of surrounding trees. A pioneer work by Hashimoto et al. (2012) demonstrated that 4 of 34 fungal RFLP types confirmed in *Pyrola asarifolia* (syn. *P. incarnata*) at one stand and 3 of 14 RFLP types at another stand were shared with coexisting *Betula* trees in the same stands (Table S3 in Hashimoto et al. 2012). Such stand scale fungal sharing, however, does not necessarily indicate physical mycorrhizal networks connecting them (Nara 2006), because the different individuals (genets) of the same fungal species detected in remote soil samples do not form physical networks for carbon transfer between the plants. Thus, it is also possible that Pyroleae plants are independently associated with ECM fungi regardless of the presence of surrounding trees, as in the autotrophic ericaceous shrubs Arbutoideae (Molina and Trappe 1982; Horton et al. 1999; Richard et al. 2005). Recently, Uesugi et al. (2016) advanced the approach of Hashimoto et al. (2012) and showed the sharing of common ECM fungi between *Pyrola japonica* and ECM trees in the same soil blocks (five of six soil blocks examined). Although the lack of the sharing could not completely deny the networks (e.g., some may extend the networks outside the sampled soil block), the fungal sharing in such a small space gives a strong evidence of the mycorrhizal networks connecting them. This approach deserves application to quantitative and detailed analyses of the mycorrhizal networks between Pyroleae and surrounding ECM trees, since ECM networks are regarded as the key to partial MH in Pyroleae and its ecological adaptation to the dark forest floor.

The colonization of diverse ECM fungi on Pyroleae roots (Tedersoo et al. 2007; Zimmer et al. 2007; Vincenot et al. 2008; Hynson and Bruns 2009; Toftegaard et al. 2010; Hashimoto et al. 2012) contrasts the high fungal specificity in closely related Monotropoideae, each of which is associated with a single fungal species or a very narrow fungal group (Bidartondo 2005 and references therein). Association with different fungi hypothetically enables coexisting Monotropoideae species to avoid competition for fungal carbon resources and niche overlap. While multiple Pyroleae species often coexist in the same forest, it is still unclear how they avoid niche overlap without differentiating mycobionts. Recently, a few Pyroleae species have been shown to associate with fungal communities biased toward a certain fungal group (Matsuda et al. 2012; Hynson et al. 2015; Uesugi et al. 2016). Such fungal preference could potentially enable sympatric niche (mycobiont community) differentiation among coexisting Pyroleae species. Although some pioneer studies have described root-colonizing fungal communities in some coexisting Pyroleae species (Tedersoo et al. 2007; Zimmer et al. 2007), no statistical comparison has been made in these descriptive studies because of the limited number of samples. Therefore, to advance our understanding of the differentiation of root-colonizing fungal communities among coexisting Pyroleae species, it is necessary to compare the communities statistically using sufficient sample sizes.

In this study, we examined root-colonizing fungal communities in the three co-existing Pyroleae species, *Pyrola alpina*, *Pyrola incarnata*, and *Orthilia secunda*, as well as ECM fungi on surrounding trees in the same soil blocks. More than 100 Pyroleae plants were evaluated, and the following hypotheses were tested: (1) ECM fungi sharing between Pyroleae plants and surrounding trees is frequent, regardless of the Pyroleae species; (2) co-existing Pyroleae species are associated with different sets of ECM fungi to avoid competition for mycobiont resources; and (3) co-existing Pyroleae species establish mycelial networks with different tree species to reduce niche overlap.

## Materials and methods

### Study site

Three forest sites on the northern side of Mt. Fuji (Table S1), Yamanashi Prefecture, Japan, were investigated. The mean annual temperature and precipitation are 3.6 °C and 2766 mm, respectively (The Japan Meteorological Agency 2016). The sites are boreal forests composed of *Abies veitchii* and *Larix kaempferi* mixed with *Salix reinii*, *Alnus sieboldiana*, and *Betula ermanii* shrubs. Three Pyroleae species, *P*. *alpina*, *P*. *incarnata*, and *O*. *secunda*, also co-exist on the forest floors (Electronic Supplementary Fig. S1).

### Sampling

Between 31 and 34 soil blocks (15 × 15 × 10 cm, length × width × depth) containing at least one Pyroleae plant per block were collected at each site in September 2013 and 2014. Pyroleae species were at least 10 m apart. In total, 95 soil blocks, including 113 Pyroleae plants, were collected (37, 44, and 32 individuals of *P*. *alpina*, *P*. *incarnata*, and *O*. *secunda*, respectively). Soil samples were collected in individual plastic bags and stored in a cool box prior to analysis.

### Root samples for molecular analyses

All roots were manually separated into Pyroleae roots (by tracing back from the plant base along rhizomes), washed with tap water, and examined using a dissecting microscope (SZX7, Olympus, Tokyo). The root tips of two *Pyrola* species had distinguishable morphological traits with obvious mantle formation or sparse extraradical hyphae (Massicotte et al. 2008; Vincenot et al. 2008) and, thus, were classified into morphotypes (Fig. S1) (Massicotte et al. 2008). Up to three root tip replicates (depending on availability) per morphotype were subjected to DNA analysis. *O*. *secunda*, however, had no obvious morphotype or mantle (Fig. S1) (Massicotte et al. 2008). Examination of *O*. *secunda* roots using 10 random root fragments from each of nine plants in 2013 revealed that fungal colonization occurred mostly in swollen lateral root regions, often accompanied by brown pigments (Fig. S1). Notably, such findings were rare in other root regions. To improve fungal detection frequency, we excised the former root regions of *O*. *secunda*, sectioned them into 2–4 mm fragments and randomly selected 4–10 fragments from each plant for fungal identification. To evaluate common mycelial networks between Pyroleae plants and surrounding trees, ECM roots from each soil block were collected and morphotyped, and fungi were identified, as described elsewhere (Murata et al. 2013). In total, 853 Pyroleae and 1273 ECM root samples were used for molecular identification.

### Fungal identification

DNA extraction and molecular identification followed that described elsewhere (Miyamoto et al. 2015). Briefly, each Pyroleae or ECM root tip was placed in a 2.0-ml tube, pulverized using a bead-beater, and DNA was extracted using a modified cetyl trimethyl ammonium bromide method. The internal transcribed spacer (ITS) regions of fungal rDNA were amplified by polymerase chain reaction (PCR) using the forward primer ITS1F and the reverse primers ITS4, LR21, LR22, and LBW with a Multiplex PCR kit (QIAGEN, Hilden, Germany), following the manufacturer’s instructions. PCR products were assessed on 1.2% agarose gels (0.5× TBE buffer) using Gel Red (Biotium, Fremont, CA, USA) under UV light (Benchtop 2UV Transilluminator, UVP, Cambridge, UK). Amplification products were purified using a PCR clean-up kit containing exonuclease I and shrimp alkaline phosphatase (GE Healthcare, Hertfordshire, UK). Sanger sequencing was performed using the Big Dye Terminator version 3.1 Cycle Sequencing kit (Applied Biosystems, Foster City, CA, USA) and ITS1 or ITS4. Sequencing was performed on the ABI 3130 XL Genetic Analyzer (Applied Biosystems). Sequences were manually corrected using the chromatograms and assembled into molecular operational taxonomic units (OTUs) using ATGC ver. 7 (GENETYX, Tokyo, Japan) with a ≥97% similarity threshold. One representative sequence for each OTU was used for BLAST searches to identify the OTU (hereafter referred to as “species” for simplicity). To identify the host tree species associated with each mycobiont, the *trnL* region of chloroplast DNA was amplified using the *trnC–trnD* and *trnE–trnF* primer pairs and then sequenced (Liu et al. 2011).

### Statistical analyses

The frequency of each fungal species was defined as the number of plants (or soil blocks) colonized by that fungus in each Pyroleae species. The relative abundance of a fungal species was defined as the percentage of Pyroleae root tips containing each fungal species. To compare fungal diversity, Jackknife2 richness estimator for each Pyroleae species was calculated with EstimateS ver. 9.1.0. (Colwell et al. 2012). To evaluate the fungal communities among plant species, non-metric multidimensional scaling (NMDS) was performed using the vegan package in R ver. 3.2.3 (R Core Team 2015). To calculate Bray–Curtis distances, 999 permutations were performed based on a fungal community matrix containing the fungal frequency data for each plant species at each site. Singleton fungal species and fungi from unidentified plant hosts were excluded prior to analysis. Statistical differences in root-associated fungal communities among sites, among Pyroleae species, and between Pyroleae and ECM trees were determined using ADONIS (permutation-based multivariate analysis of variance). A total of 999 permutations were performed for all such analyses.

### Network architecture

The architecture of mycobiont sharing was visualized based on the Fruchterman-Reingold algorithm (Fruchterman and Reingold 1991) implemented in the igraph package for R (Ognyanova 2015), using the mycobiont sharing data between each Pyroleae species and surrounding ECM trees within the same soil blocks. The same analysis was performed for the data set including all plant-fungal occurrence in three study sites for visualizing land scape level fungal sharing (Toju et al. 2013), although it does not necessarily indicate direct (physical) mycorrhizal networks for MH carbon transfer.

## Results

### General description of the root-associated fungal community in Pyroleae and ectomycorrhizal trees

A total of 180 putative mycorrhizal fungal species were identified from the roots of Pyroleae and surrounding trees. Basidiomycetes were dominant (164 spp.), while 16 species (9%) belonged to ascomycetes (Table S2). The Jackknife2 estimator of species richness was 307.

### Mycobionts in *O. secunda*

**Fig. 1:**
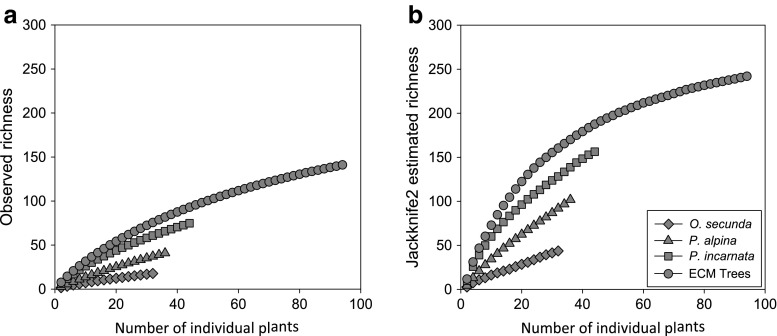
Species accumulation curves of **a** observed species richness and **b** estimated richness (Jackknife2) for each Pyroleae species and all ectomycorrhizal trees coexisting on Mount Fuji, Japan

Forty-eight (33.8%) of 142 root tips from 32 plants were sequenced. Mycobionts were not identified from 12 plants. In total, 18 fungal species were identified using a >97% ITS similarity threshold, and ~44 species were identified in *O*. *secunda* roots using Jackknife2 (Table 1, Fig. 1 and Table S2). The most frequent lineage in *O*. *secunda* roots was *Wilcoxina*, which was found in 22 of 48 root fragments and in 12 of 20 plant individuals (Tables 2 and S2). *Phialocephala* was the second most frequent lineage, found in four root fragments from four plants. *Russula* and *Sebacina* were detected once in each of three plant individuals. The fungal richness (mean ± SE) in *O*. *secunda* roots per soil block was 1.6 ± 0.2 or 1.0 ± 0.2 if uncolonized plants were included (Table 1).

**Table 1:** Fungal communities on Pyroleae and dominated tree species on Mount Fuji, Japan

Plant species	Occurrence in soil block	Number of root tips	Fungal richness per soil block^a^	Observed fungal richness	Jackknife2	Shannon’s H′	Simpson’s 1/D	ECM richness per soil block
*O. secunda*	32	48 (142)	1.0 ± 0.2 a	18	44	2.6	10.1	4.0 ± 0.4
*P. alpina*	37	95 (233)	1.8 ± 0.2 a	42	105	3.6	30.9	4.4 ± 0.2
*P. incarnata*	44	307 (483)	3.4 ± 0.2 b	75	156	4	33.3	3.6 ± 0.1
Host trees								
Totals in ECM tips		768 (1267)		142	243	4.4	44.1	
*Larix kaempferi*	70			69				
*Abies veitchii*	47			73				
*Betula ermanii*	33			44				
Others				34				

^a^Significant difference in fungal richness (*P* < 0.05) among Pyroleae species was indicated by different lowercase letters

**Table 2:** Preference and antagonism of three coexisting Pyroleae species toward fungal lineages with reference to coexisting ectomycorrhizal fungi across three sites on Mount Fuji, Japan

	Soil blocks containing *O. secunda*		Soil blocks containing *P. alpina*		Soil blocks containing *P. incarnata*	
Lineage	*O. secunda*	ECM Trees	*P. alpina*	ECM Trees	*P. incarnata*	ECM Trees
*Cortinarius*	3^a^	37	14	36	55	53
Thelephoraceae	1	20	7	19	37*	20
*Cenococcum*	0	15	1	20	1**	17
*Russula*	3	9	3	10	9	10
*Inocybe*	0	9	4	11	9	8
*Lactarius*	0	14	1	13	0	10*
*Hygrophorus*	1	2	2	10	1	7
*Sebacina*	3	2	4	1	8	3
*Amphinema*	2	0	8**	2	9	3
*Wilcoxina*	12**^,b^	0	0	1	0	2
*Suillus*	0	4	0	6	0	5
*Phialocephala*	4**	0	1	0	1	1
Other	2	19	13	27	20	22
Total mycobionts	31	132	57	156	150	160

^a^Cumulative number of soil blocks containing each fungal species, regarded as the same as the total number of fungal individuals (genets or mycobionts in the text) belonging to that species, was pooled for individual fungal lineages

^b^Significant difference in occurrence at *p* < 0.01 (*) and *p* < 0.001 (**) between Pyroleae and ectomycorrhizal trees based on Fisher’s exact tests. To reduce the possibility of false positive in multiple tests, strict *p* values were adopted here

*p < 0.01; **p < 0.001

### Mycobionts in *P. alpina*

Ninety-five (40.8%) of 233 root tips from 37 plants were sequenced. These sequences were grouped into 42 species (~105 using Jackknife2; Table 1 and Fig. 1), including the diverse ECM fungal lineages. *Cortinarius*, *Amphinema*, and *Thelephora*/*Tomentella* were found in 11, 8, and 5 *P*. *alpina* individuals, respectively (Table 2, Table S2). Other ECM fungal genera, including *Sebacina*, *Russula*, *Inocybe*, *Hebeloma*, *Hygrophorus*, and *Piloderma*, were detected in two to four plant individuals. Most mycobionts belonged to Basidiomycetes, while only two belonged to Ascomycetes. The fungal richness in *P*. *alpina* roots per soil block was 1.8 ± 0.2 (Table 1).

### Mycobionts in *P. incarnata*

**Fig. 2:**
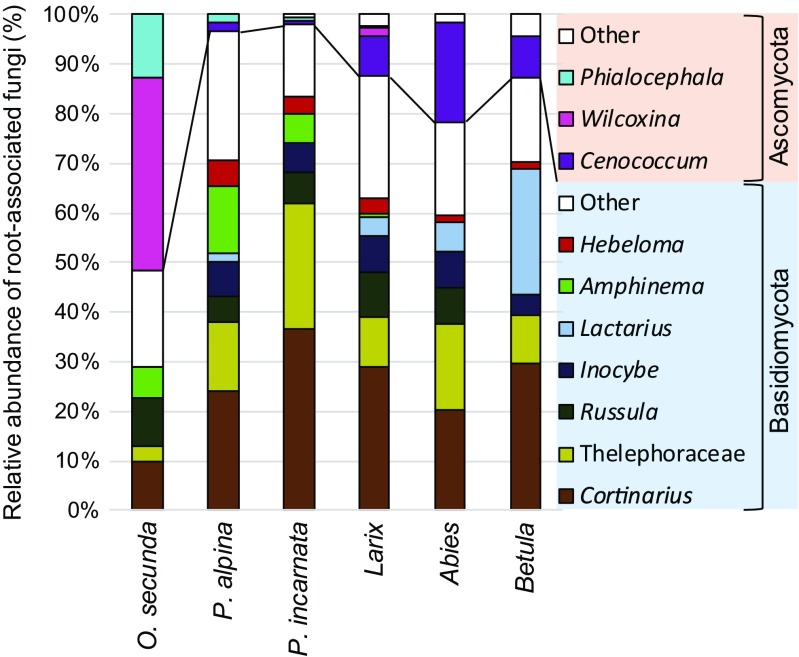
Relative abundances of fungal lineages associated with roots of Pyroleae and ectomycorrhizal trees coexisting in subalpine forests on Mount Fuji, Japan. Data from Table S2

Three hundred and seven (63.6%) of 483 tips from 44 plants were sequenced and grouped into 75 fungal species (~156 using Jackknife2, Table 1). Similar to *P*. *alpina*, mycobionts in *P*. *incarnata* roots were dominated by the ECM fungal genera *Cortinarius* and *Thelephora*/*Tomentella* (Table 2, Table S2), which were detected in 29 plants. Other frequently observed genera were *Laccaria*, *Amphinema*, *Sebacina*, *Russula*, *Inocybe*, and *Hebeloma*, each of which was found in five to nine plants. As in *P*. *alpina*, Basidiomycetes ECM fungi were dominant (Fig. 2). The fungal richness in *P*. *incarnata* roots per soil block was 3.4 ± 0.2, which was significantly higher than that in the other two Pyroleae species (*p* < 0.05, Tukey’s test; Table 1).

### Ectomycorrhizal fungi on surrounding trees in Pyroleae-containing soil blocks

Of 1267 ECM tips from surrounding trees, 768 (61%) were sequenced. In total, 142 fungal species (243 using Jackknife2) were identified from a total of 95 soil blocks. The dominant ECM fungi were *Cortinarius* (59 soil blocks), *Russula*/*Lactarius* (47 blocks), *Cenococcum* (42 blocks), and *Thelephora*/*Tomentella* (42 blocks), followed by *Inocybe* (23 blocks), *Suillus* (14 blocks), *Piloderma* (12 blocks), *Laccaria* (11 blocks), *Hygrophorus* (10 blocks), *Hebeloma* (8 blocks), and *Sebacina* (5 blocks) (Table S2). *Clavulina*, *Amphinema*, and *Amanita* were each found in three or fewer soil blocks. Using chloroplast DNA sequences, host tree species were identified in 85% of ECM tips. *Larix*, *Abie*s, and *Betula* were dominant ECM hosts, found in 70, 47, and 33 soil blocks, respectively (Table 1). The ECM fungal richness values (mean ± SE) per soil block containing *O*. *secunda*, *P*. *alpina*, and *P*. *incarnata* were 4.0 ± 0.4, 4.4 ± 0.2, and 3.6 ± 0.1, respectively (*p* > 0.05, Table 1).

### Mycorrhizal fungal communities among Pyroleae and canopy trees on a landscape scale

Of 180 fungal species identified, 66 were found in both Pyroleae and ECM trees, after pooling all soil blocks from the three sites. These common species on a landscape scale included 12 of 18, 26 of 42, and 53 of 75 fungal species in *O*. *secunda*, *P*. *alpina*, and *P*. *incarnata* roots, respectively (Table S2). *Cenococcum* and *Lactarius* were rarely confirmed in Pyroleae roots, in contrast to their abundance on ECM tips of the surrounding trees (Fig. 2 and Table 2).

**Fig. 3:**
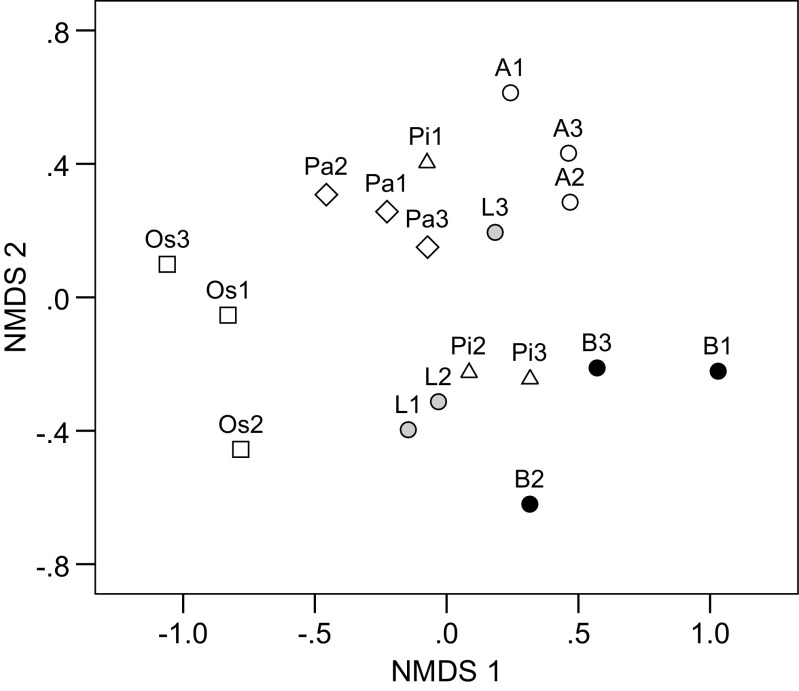
Non-metric multidimensional scaling plot showing similarity in root-associated fungal communities of Pyroleae and surrounding ectomycorrhizal trees on Mount Fuji, Japan. Hosts represented by <six soil samples were excluded before the analyses. Bray-Stress = 0.184. *Circles* represent the communities on ectomycorrhizal trees, where *Abies* (*A*), *Betula* (*B*), and *Larix* (*L*) are in *white*, *black*, and *gray*, respectively. Other *open symbols* represent the communities on Pyroleae spp., where *Orthilia secunda* (*Os*), *Pyrola alpina* (*Pa*), and *Pyrola incarnata* (*Pi*) are in *squares*, *diamonds*, and *triangles*, respectively. Site numbers are shown after host abbreviations

We used NMDS and ANONIS analyses to evaluate the differences in root-associated fungal communities among Pyroleae and ECM tree species. After excluding the minor hosts (i.e. <5 soil blocks per site, singleton fungal species, fungi on unidentified hosts), the data set used for the community analyses contained 107 fungal species. Fungal communities were separated by plant species rather than by sites in the NMDS plot (Fig. 3). Fungal communities of *O*. *secunda* were separated from those of other plant species. Fungi in *P*. *alpina* roots were partially separated from those in ECM trees, with the exception of *Larix* in Site 3. Additionally, fungal communities of *P*. *incarnata* were in close proximity to those of ECM trees at the same site.

The ADONIS test confirmed that the data variance in the fungal communities was significantly explained by plant species (*F*_5, 12_ = 2.44, *r* ^2^ = 0.50, *p* = 0.001), while the effect of sites was not significant (*F*_2, 15_ = 1.02, *r* ^2^ = 0.12, *p* = 0.417). We further compared the fungal communities among Pyroleae species, after excluding ECM trees and found a significant difference among Pyroleae species (*F*_2, 6_ = 2.68, *r* ^2^ = 0.47, *p* = 0.007), but not among sites (*F*_2, 6_ = 0.67, *r* ^2^ = 0.18, *p* = 0.833).

While the occurrence of common fungal species between Pyroleae and ECM trees on the landscape scale does not necessarily indicate direct physical connection for MH carbon transfer, *P*. *incarnata* is connected to ECM trees by many fungal species in a landscape-scale network plot (Fig. S2). In contrast, *O*. *secunda* and ECM trees had far fewer common fungal species across the study sites (Fig. S2).

### Mycobiont sharing between Pyroleae and canopy tree species on a soil block scale

**Fig. 4:**
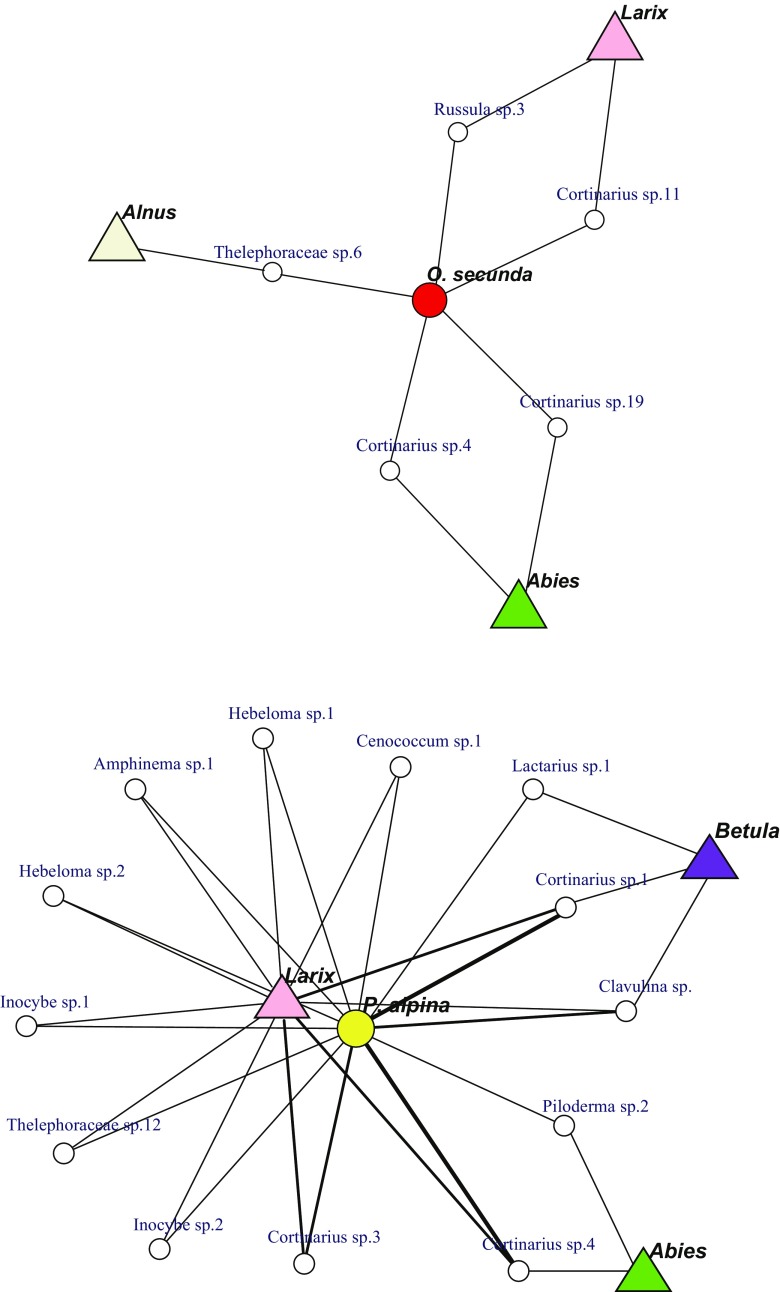

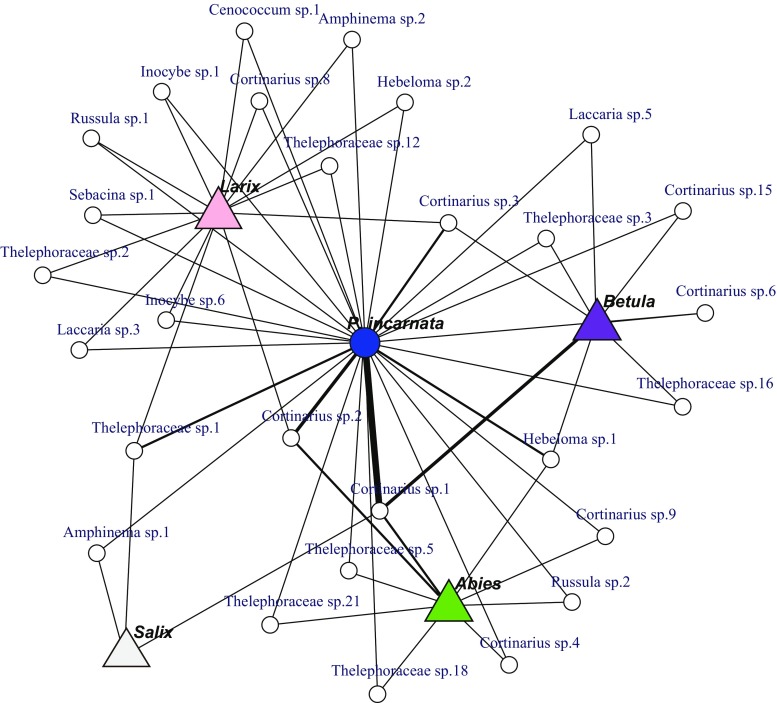
Confirmed mycobiont sharing between Pyroleae and ectomycorrhizal (ECM) trees within the same soil blocks collected in subalpine forests on Mount Fuji, Japan. **a** *Orthilia secunda*. **b** *Pyrola alpina*. **c** *Pyrola incarnata*. Data from Table 3. *White circles* represent mycobiont species confirmed in roots of both Pyroleae and ECM trees within the same soil blocks. *Line width* is proportional to the number of occurrence, i.e., the number of soil blocks or plant individuals that contained the fungus. Because of the sharing within the small space (*soil block*), *line connections* between plants in these panels strongly suggest physical networks enabling MH carbon transfer. Note that very few thin connections to ECM trees from *O. secunda* but many (and some thick) connections from *Pyrola incarnata*

Mycobiont sharing between Pyroleae and canopy trees was defined as the presence of the same fungal species in both plant groups within a soil block, assuming common mycorrhizal networks between them. In *O*. *secunda*, mycobiont sharing was rare, as it was confirmed in only 5 of 31 (16.1%) mycobionts in *O*. *secunda* roots and in 5 of 32 *O*. *secunda* individuals (Table 3, Fig. 4a). Three canopy tree genera (*Larix*, *Abies*, and *Alnus*) shared mycobionts with *O*. *secunda*, although they represented only 5 of 51 ECM trees in the same blocks (Table 3, Fig. 4a). Of the five shared mycobionts, three belonged to *Cortinarius*. Although *Wilcoxina* was the most dominant mycobiont lineage in *O*. *secunda* roots (Table 2, Fig. S2), it was not confirmed in any of the surrounding ECM tips within the same soil blocks (*p* < 0.001).

**Table 3:** Mycobiont sharing between Pyroleae and canopy trees in the same soil blocks

	*O. secunda*	*P. alpina*	*P. incarnata*
Shared mycobionts (total mycobionts)^a^	5 (31)	19 (57)	44 (150)
Shared plants (total)^b,^**	5 (32)	12 (37)	30 (44)
With Larix*	2 (18)	10 (28)	19 (36)
With Abies**	2 (20)	2 (15)	10 (16)
With *Betula*	0 (10)	3 (13)	6 (15)
With *Salix*	0 (0)	0 (1)	2 (3)
With *Alnus*	1 (3)	0 (5)	1 (4)
Fungal species shared with coexisting tree^c^			
*Amphinema* sp.1		L(1)	S(1)
*Amphinema* sp.2			L(1)
*Cenococcum* sp.1		L(1)	L(1)
*Clavulina* sp.		L(1), B(1)	
*Cortinarius* sp.1		L(2), B(1)	L(7), A(2), B(3), S(1)
*Cortinarius* sp.2			L(1), A(2)
*Cortinarius* sp.3		L(2)	L(1), B(1)
*Cortinarius* sp.4	A(1)	L(2), A(1)	A(1)
*Cortinarius* sp.6			B(1)
*Cortinarius* sp.8			L(1)
*Cortinarius* sp.9			A(1)
*Cortinarius* sp.11	L(1)		
*Cortinarius* sp.15			B(1)
*Cortinarius* sp.19	A(1)		
*Hebeloma* sp.1		L(1)	A(1), B(1)
*Hebeloma* sp.2		L(1)	L(1)
*Inocybe* sp.1		L(1)	L(1)
*Inocybe* sp.2		L(1)	
*Inocybe* sp.6			L(1)
*Laccaria* sp.3			L(1)
*Laccaria* sp.5			B(1)
*Lactarius* sp.1		B(1)	
*Piloderma* sp.2		A(1)	
*Russula* sp.1			L(1)
*Russula* sp.2			A(1)
*Russula* sp.3	L(1)		
*Sebacina* sp.1			L(1)
Thelephoraceae sp.1			L(1), S(1)
Thelephoraceae sp.2			L(1)
Thelephoraceae sp.3			B(1)
Thelephoraceae sp.5			A(1)
Thelephoraceae sp.6	Aj(1)		
Thelephoraceae sp.12		L(1)	L(1)
Thelephoraceae sp.16			B(1)
Thelephoraceae sp.18			A(1)

*L Larix*, *A Abies*, *B Betula*, *S Salix*, *Aj Alnus*

*Statistically significant difference in the proportion of fungal shared plants at *p* < 0.01 among Pyroleae species based on Fisher’s exact test

**Statistically significant difference in the proportion of fungal shared plants at *p* < 0.001 among Pyroleae species based on Fisher’s exact test

^a^Occurrence of the same fungal species in multiple roots from a single Pyroleae plant should be regarded as a single mycobiont individual (a fungal genet), while the occurrence in different soil blocks could be treated as different mycobionts. Thus, in this table, the number of mycobionts shared with coexisting ectomycorrhizal trees in the same soil block is shown, followed by the total number of mycobionts confirmed in each Pyroleae species

^b^The number of Pyroleae individuals sharing at least one common mycobiont with coexisting trees in the same soil block was shown, followed by the total number of plant individuals sampled. Shared tree species are described in the following lines, where the number of shared trees is followed by the total number of trees coexisted with each Pyroleae species in the same soil blocks

^c^Shared fungal species are listed with the information of shared tree species. Note that fungal sharing with multiple tree species was observed in some soil blocks. Taxonomic labels of fungi are as in Table S1

In *P*. *alpina*, sharing was confirmed in 12 of 37 plants by 19 of 57 (33.3%) mycobiont individuals (Table 3). Three canopy tree genera, including *Larix*, *Abies*, and *Betula*, shared mycobionts with *P*. *alpina* (Fig. 4b), accounting for 16 of 61 ECM trees confirmed in the same blocks (Table 3). Of 19 mycobionts (13 species) shared with surrounding ECM trees, 8 (three species), 2, and 2 belonged to *Cortinarius*, *Hebeloma*, and *Inocybe*, respectively (Table 3, Fig. 4b). Although *Amphinema* was the second most dominant mycobiont lineage in *P*. *alpina* (Table 2), it was not confirmed in surrounding ECM tips within the same blocks (*p* < 0.001, Fig. S2). Conversely, *Cenococcum* was frequently found in the surrounding ECM tips but rarely found in *P*. *alpina* roots (Table 2, Fig. S2).

Mycobiont sharing with canopy trees was most frequently observed in *P*. *incarnata* (Fig. 4c). Such sharing was confirmed in 30 of 44 plant individuals by 44 of 150 (29.3%) mycobionts (Table 3). Of 44 mycobiont individuals (27 species) shared with surrounding canopy trees, 23 (8 species) and 8 (8 species) belonged to *Cortinarius* and Thelephoraceae, respectively (Table 3). Trees sharing common mycobionts with *P. incarnata* included *Larix*, *Abies*, *Betula*, and *Salix* (Fig. 4c), collectively representing more than half of the trees detected within the same soil blocks. Similar to *P*. *alpina*, *Cenococcum* was rarely found in *P*. *incarnata* roots; however, it was frequently found in the surrounding ECM tips (*p* < 0.001, Table 2). The occurrence of *Lactarius* was also negatively biased against *P*. *incarnata* (*p* < 0.01, Table 2). Conversely, Thelephoraceae was more prevalent in *P*. *incarnata* roots, compared with its prevalence in the surrounding ECM tips (*p* < 0.01, Table 2).

The occurrence of mycobiont sharing was significantly different among Pyroleae species (Fig. 4a–c). The lowest proportion of sharing was found in *O*. *secunda*, while the highest was found in *P*. *incarnata* (Table 3, *p* < 0.001, Fisher’s exact test). This was further confirmed by network plots (Fig. 4), where there are few connections between *O*. *secunda* and ECM trees but many connections between *P*. *incarnata* and ECM trees within the same soil blocks.

While *Larix*, *Abies*, and *Betula* roots coexisted with all Pyroleae species, the proportion of shared trees was significantly different among Pyroleae (Table 3). For example, of the 18 *Larix* trees coexisting with *O*. *secunda*, only 2 were shared with common mycobionts. The proportion of *Larix* trees shared with *P*. *incarnata* was much higher (*p* = 0.009, Table 3). Such differences in the shared tree proportion among Pyroleae species were also observed in *Abies* (*p* = 0.001) (Table 3).

## Discussion

Root-associated fungi in three coexisting Pyroleae species were dominated by putative ECM fungal lineages, most of which have been confirmed in ECM trees in this study or in previous studies on Mt. Fuji (Nara et al. 2003; Miyamoto et al. 2014; Miyamoto et al. 2015). The dominance of ECM fungal lineages has been repeatedly shown in previous studies as mentioned in the “Introduction.” Most of these studies assumed common mycorrhizal networks linking Pyroleae and surrounding ECM trees; however, such networks have not been rigorously tested. Only one study documented the actual sharing of mycobionts between *P*. *japonica* and surrounding trees in five of six soil blocks (Uesugi et al. 2016), but the small sample size needed further confirmation. In the current study, *P*. *incarnata* shared common mycobionts with surrounding trees in nearly 70% of soil blocks, indicating that common mycorrhizal networks are common in this species. However, mycobiont sharing was confirmed in only 15% of soil blocks containing *O*. *secunda* and in 32% soil blocks containing *P*. *alpina*. The limited sharing in *O*. *secunda* was also confirmed in the analyses based on mycobiont individuals, i.e., only 16% mycobionts confirmed in *O*. *secunda* (yet all of them were minor, potentially indicating surface adhesion) was shared with surrounding trees, while about 30% mycobionts in two *Pyrola* species (many of them were dominant colonizers detected in multiple roots) were shared with the trees. Such an observation contradicts our first hypothesis. Since common mycorrhizal networks are nearly ubiquitous among ECM trees of the same species (Lian et al. 2006) and between different ECM trees (Nara and Hogetsu 2004; Nara 2006), the lower frequencies of mycobiont sharing between coexisting pairs were unexpected. Thus, some Pyroleae species, especially *O*. *secunda* in this study, may avoid sharing common mycobionts with surrounding trees and possibly maintain independent associations with ECM fungi. While the detection of ECM fungi in Pyroleae roots has been regarded as evidence of mycorrhizal networks with surrounding trees, we should be cautious about such assumptions.

Given the methodological limitations of this study (e.g. small soil blocks and small root sections for DNA analyses), it is difficult to completely exclude the potential for mycorrhizal networks between Pyroleae and ECM trees by the lack of their detection. Indeed, the fungal sharing data documented here would surely be underestimates due to undetected minor colonization, potential networks beyond the soil blocks, and ephemeral connections in other seasons. However, the lower frequencies of mycobiont sharing in *O*. *secunda* or *P*. *alpina*, compared with that of *P*. *incarnata*, are irrelevant to such methodological limitations, since the same sampling and analytical methods were used throughout this study. Similarly, the number of colonized roots was lower in *O*. *secunda* and *P*. *alpina* compared with *P*. *incarnata*. We are not sure how such a lower frequency of mycobiont sharing and lower rate of fungal colonization in *O*. *secunda* could effectively function in exploring for fungal carbon or in being epiparasitic to surrounding trees for carbon. Less morphological differentiation in fungus-colonized roots of *O*. *secunda* (diffuse roots with no fungal mantle; Fig. S1) may also be indicative of the limited carbon dependency on fungi, since full MH relatives (Monotropoideae) exhibit considerable morphological root differentiation (e.g. a “root ball” composed of a dense cluster of fungus-colonized short roots with well-developed mantles). While the root system of *P*. *incarnata* was still diffuse compared with that of Monotropoideae, fungus-colonized roots had well-developed fungal mantles (Fig. S1). Collectively, mycoheterotrophic levels may be high in *P*. *incarnata* but low or absent in *O*. *secunda*. Interestingly, our data regarding stable isotope signatures and photosynthetic traits examined within the same sites also support this hypothesis (Jia et al. in preparation).

Root-associated ECM fungi detected in two *Pyrola* species were diverse; however, the frequency of *Cenococcum* in both *Pyrola* species was significantly less than that of surrounding ECM tips. Similarly, the frequency of *Lactarius* was also lower in both *Pyrola* species but not statistically different (*p* = 0.119) in *P*. *alpina*. Antagonism toward Ascomycetes and *Lactarius* was suggested by Hashimoto et al. (2012) and was statistically confirmed, for the first time, by this study. Conversely, preferences toward specific fungal lineages were also statistically confirmed in the two *Pyrola* species (e.g. Thelephoraceae in *P*. *incarnata* and *Amphinema* in *P*. *alpina*). Compared with the two *Pyrola* species, *O*. *secunda* was associated with fewer fungal lineages and was dominated exclusively by *Wilcoxina*. The colonization of *Wilcoxina* in *O*. *secunda* roots has been documented in previous studies, but in one or two plants mainly due to the limited number of samples used (Tedersoo et al. 2007; Zimmer et al. 2007). *Wilcoxina* forms ectendomycorrhiza on conifer and broadleaf trees in nurseries, burned sites, and disturbed sites (Yang and Korf 1985; Egger 1996), but its ecology in old growth forests is unknown.

The occurrence of *Wilcoxina* on *O*. *secunda* roots and not on the surrounding ECM roots suggests an independent symbiotic association of *O*. *secunda* with *Wilcoxina*. Because *Wilcoxina* mycelium is described as “short distance exploration type” (Agerer 2001; Smith and Read 2008), its mycelia would less likely to extend beyond the soil block to form networks without colonizing nearby ECM roots. Moreover, in Tedersoo et al. (2007), 10 of 19 fungal species detected in *O*. *secunda* roots were non-ECM fungal lineages, in which root endophytes like *Phialocephala* and Helotiales were far more abundant than ECM fungi in relative abundance. This corresponds with our results and may imply less ECM networks between *O*. *secunda* and surrounding trees even in Europe, yet rigorous tests like our approach would be needed before conclusion. In fact, the mycoheterotrophy levels for *O*. *secunda* estimated from ^13^C signatures are inconsistent among sites, suggesting a lack of tripartite networks in some locations (Tedersoo et al. 2007; Zimmer et al. 2007; Johansson et al. 2015).

With contrasting fungal preference/antagonism, the three co-existing Pyroleae species were associated with significantly different communities of root-colonizing fungi. Although the two *Pyrola* species were associated with similar ECM fungal lineages, they differed in mycobiont sharing patterns. *P*. *incarnata* was incorporated into surrounding ECM networks intensively, whereas *P*. *alpina* was not so intensively. These data support our second hypothesis and suggest that the co-existing Pyroleae species avoid niche overlaps by associating with different fungi (Bever et al. 2010). Given the higher frequency of mycobiont sharing among *P*. *incarnata* with surrounding trees, carbon supply from surrounding trees through mycorrhizal networks would be critical in this species (Tedersoo et al. 2007; Zimmer et al. 2007; Matsuda et al. 2012). By contrast, the lower frequency of mycobiont sharing and association with unique fungal communities in *O*. *secunda* raise doubts on carbon supply from surrounding trees in some locations. *O*. *secunda* may seek other resources, such as nitrogen, from root-associated fungi (Hynson et al. 2013b).

The few connections to surrounding trees in *O*. *secunda* partly collapse our third hypothesis. Yet, the two *Pyrola* species were associated with slightly different proportions of the surrounding trees, i.e., *P. incarnata* was connected to *Abies*, *Betula*, and *Larix* rather evenly (Table 3, Fig. 4c), while *P. alpina* shared common mycotionts with *Larix* mostly (Table 3, Fig. 4b). Thus, the two *Pyrola* species may partly avoid niche overlaps by differentiating host trees for sharing, in addition to the different abundance of the sharing.

Pyroleae is a unique plant lineage that includes autotrophic (Zimmer et al. 2007), partial MH (Tedersoo et al. 2007; Zimmer et al. 2007), and full MH (Hynson and Bruns 2009) species and provides an opportunity to investigate its evolution from an autotrophic to full MH species (Lallemand et al. 2016). In Orchidaceae, the evolution of full MH species is hypothesized to occur with changes in root-associated fungi from the saprotrophic *Rhizoctonia* to ECM fungi and from a broad to narrow range of mycorrhizal fungi (Leake 2004; Bidartondo 2005; Selosse and Roy 2009; Motomura et al. 2010; Ogura-Tsujita et al. 2012). However, full MH *Pyrola aphylla* was associated with a broad range of ECM fungi, suggesting that the situation in Orchidaceae is not applicable to the evolution of mycoheterotrophy in Pyroleae (Hynson and Bruns 2009). While most previous studies on Pyroleae mycobiont communities are descriptive based on fewer samples, the quantitative and community analyses based on enough samples including surrounding ECM roots would be necessary for detecting inconspicuous but significant preference or antagonism to certain fungal lineage. The accumulation of such data in other geographical locations and other Pyroleae species would advance our understanding about the evolution of their mycobiont associations, and its geographical variance or local adaptation.

## Electronic supplementary material

Supplementary Fig S1 (DOCX 412 kb)

Supplementary Fig S2 (DOCX 412 kb)

Supplementary Table S1 (DOCX 13 kb)

Supplementary Table S2 (DOCX 63 kb)

Supplementary Table S3 (DOCX 22 kb)
