# Supplementary material for: Root-associated fungal communities in three Pyroleae species and their mycobiont sharing with surrounding trees in subalpine coniferous forests on Mount Fuji, Japan
Source: Mycorrhiza. 2017 Jul 13;27(8):733–45. doi: 10.1007/s00572-017-0788-6 (PMC5645451; doi:10.1007/s00572-017-0788-6)
Supplement: Supplementary file 1 — (DOCX 412 kb) [file 572_2017_788_MOESM1_ESM.docx]

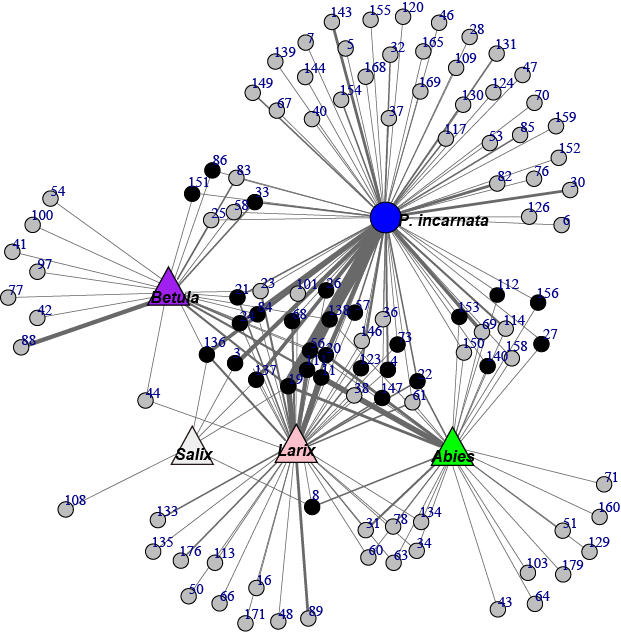

**Fig. S2** Network plots visualizing the frequency of common fungal species between Pyroleae species and surrounding ectomycorrhizal (ECM) trees after pooling samples for each Pyroleae species across three study sites on Mount Fuji, Japan. (a) *Orthilia secunda*, (b) *Pyrola alpina*, or (c) *Pyrola incarnata*. Fungal species are shown in black (confirmed mycobiont sharing within the same soil blocks as in Fig.4) or grey circles (shared but in different soil blocks across the sites) with ID numbers in Table S2. Line width is proportional to the number of occurrence, i.e. the number of soil blocks or plant individuals containing each fungus. The network plots after pooling all the three Pyroleae samples (all soil blocks) is shown in (d) and (e), the latter of which is grouped into ECM fungal lineages shown in Table S3 (lineage ID No. available). In the (e) panel, ascomycetes and basidiomycetes are shown in grey and black, respectively. The line width is shown as one fifth of the number of occurrence in the (e) panel. Note that line connections between plants in all these panels do not necessarily indicate physical connections by common mycobionts within the same soil blocks.
