# Supplementary material for: Root-associated fungal communities in three Pyroleae species and their mycobiont sharing with surrounding trees in subalpine coniferous forests on Mount Fuji, Japan
Source: Mycorrhiza. 2017 Jul 13;27(8):733–45. doi: 10.1007/s00572-017-0788-6 (PMC5645451; doi:10.1007/s00572-017-0788-6)
Supplement: Supplementary file 3 — (DOCX 13 kb) [file 572_2017_788_MOESM3_ESM.docx]

| **Table S1** Description of study sites on Mt. Fuji | | | | |
| --- | --- | --- | --- | --- |
| Site | S1 | S2 | S3 |  |
| Elevation (m) | 2259 | 2270 | 2246 |  |
| Coordinates | N35º23.450' | N35º23.512' | N35º23.445' |  |
|  | E138º43.154' | E138º43.315' | E138º42.863' |  |
| Temperature (ºC)^1^ |  | 3.6 |  |  |
| Precipitation (mm)^1^ |  | 2766 |  |  |
| Tree density (stems per ha) |  | 1477 |  |  |
| Number of host tree species |  | 5 |  |  |
| pH (H_2_O)^2^ | 4.0-5.9 (4.9) | 4.0-5.8 (4.9) | 4.0-6.2 (4.9) |  |
| ^1^ Site-specific mean annual temperature and mean annual precipitation obtained from the interpolated mesh data (1 × 1km^2^; 30-year averages from 1981-2010) provided by the Japan Meteorological Agency, 2016. | | | | |
| ^2^ Values are minimum–maximum with the mean in parentheses. | | | | |
