# Supplementary material for: Root-associated fungal communities in three Pyroleae species and their mycobiont sharing with surrounding trees in subalpine coniferous forests on Mount Fuji, Japan
Source: Mycorrhiza. 2017 Jul 13;27(8):733–45. doi: 10.1007/s00572-017-0788-6 (PMC5645451; doi:10.1007/s00572-017-0788-6)
Supplement: Supplementary file 4 — (DOCX 63 kb) [file 572_2017_788_MOESM4_ESM.docx]

| **Table S2** Root associated fungal species found in three Pyroleae species and coexisting ectomycorrhizal trees on Mount Fuji, Japan | | | | | | | | | | | | |
| --- | --- | --- | --- | --- | --- | --- | --- | --- | --- | --- | --- | --- |
| ID  No. | Fungal species^1^ | Pyroleae^2^ | | | Ectomycorrhizal trees^3^ | | | | Accession no. | Seq. length (bp) | Best blastn match | |
|  |  | *Os* | *Pa* | *Pi* | *L* | *A* | *B* | O |  |  | Accession no. | Iden (%) |
| 1 | *Alnicola* sp. |  |  |  | 1 |  |  | 1 | LC276955 | 365 | JN943941.1 | 1 |
| 2 | *Amanita* sp. |  |  |  |  | 4 |  |  | LC276956 | 626 | JF907764.1 | 0.99 |
| 3 | *Amphinema* sp. 1 |  | 4 | 7 | 1 |  |  | 1 | LC276957 | 705 | GQ162811.1 | 0.98 |
| 4 | *Amphinema* sp. 2 | 2 | 4 | 3 | 1 |  |  |  | LC276958 | 702 | GQ162811.1 | 0.88 |
| 5 | *Aphyllophorales* sp. | 1 |  | 1 |  |  |  |  | LC276959 | 578 | EF619631.1 | 0.99 |
| 6 | Ascomycota sp. 1 |  |  | 1 |  | 1 |  |  | LC276960 | 364 | AY268199.1 | 0.98 |
| 7 | Ascomycota sp. 2 |  | 1 | 1 |  |  |  |  | LC276961 | 426 | HQ211785.1 | 0.98 |
| 8 | *Boletus* sp. 1 |  |  |  | 2 | 4 |  |  | LC276962 | 782 | AB821457.1 | 0.99 |
| 9 | *Boletus* sp. 2 |  |  |  |  | 1 |  |  | LC276963 | 738 | AB848410.1 | 0.99 |
| 10 | *Boletus* sp. 3 | 1 | 1 |  |  |  |  | 1 | LC276964 | 686 | JQ991667.1 | 0.99 |
| 11 | *Cenococcum* sp. 1 |  | 1 | 1 | 14 | 18 | 5 | 3 | LC276965 | 497 | KF879454.1 | 0.99 |
| 12 | *Cenococcum* sp. 2 |  |  |  |  | 1 | 1 | 1 | LC276966 | 419 | FJ378838.1 | 0.99 |
| 13 | *Cenococcum* sp. 3 |  |  |  |  | 3 |  |  | LC276967 | 189 | DQ474390.1 | 0.99 |
| 14 | *Cenococcum* sp. 4 |  |  |  |  | 1 |  |  | LC276968 | 298 | AB848418.1 | 1 |
| 15 | *Cantharellales* sp. |  | 1 |  |  |  |  |  | LC276969 | 295 | DQ990870.1 | 0.99 |
| 16 | *Chroogomphus* sp. |  |  |  | 1 |  |  |  | LC276970 | 301 | JX029937.1 | 0.96 |
| 17 | *Clavulina* sp. |  | 1 |  | 1 | 1 | 1 |  | LC276971 | 619 | JX198537.1 | 0.96 |
| 18 | *Clitocybe* sp. |  | 1 |  |  |  |  |  | LC276972 | 578 | JF907817.1 | 0.86 |
| 19 | *Cortinarius* sp. 1 |  | 6 | 17 | 13 | 4 | 4 | 6 | LC276973 | 838 | FJ717538.1 | 0.99 |
| 20 | *Cortinarius* sp. 2 |  | 3 | 6 | 5 | 4 | 2 | 3 | LC276974 | 639 | GQ159884.1 | 0.95 |
| 21 | *Cortinarius* sp. 3 |  | 2 | 2 | 5 | 4 | 1 | 1 | LC276975 | 784 | FJ039700.1 | 0.98 |
| 22 | *Cortinarius* sp. 4 | 1 | 3 | 2 | 5 | 4 |  |  | LC276976 | 655 | DQ481834.1 | 0.99 |
| 23 | *Cortinarius* sp. 5 |  |  | 1 | 8 |  | 1 | 1 | LC276977 | 707 | HQ604705.1 | 0.99 |
| 24 | *Cortinarius* sp. 6 |  |  | 5 | 1 |  | 2 | 1 | LC276978 | 851 | FJ039708.1 | 0.99 |
| 25 | *Cortinarius* sp. 7 |  |  | 1 | 2 |  | 2 |  | LC276979 | 765 | FN669182.1 | 0.99 |
| 26 | *Cortinarius* sp. 8 |  |  | 4 | 1 |  |  | 1 | LC276980 | 642 | HQ604726.1 | 0.98 |
| 27 | *Cortinarius* sp. 9 |  |  | 1 |  | 3 |  |  | LC276981 | 740 | GQ159898.1 | 0.99 |
| 28 | *Cortinarius* sp. 10 |  |  | 1 |  |  | 1 | 1 | LC276982 | 821 | HQ604694.1 | 0.98 |
| 29 | *Cortinarius* sp. 11 | 1 |  |  | 1 | 1 |  |  | LC276983 | 643 | HQ604699.1 | 0.99 |
| 30 | *Cortinarius* sp. 12 |  |  | 4 |  | 1 | 1 |  | LC276984 | 496 | HQ604732.1 | 0.99 |
| 31 | *Cortinarius* sp. 13 |  |  |  | 1 | 4 |  |  | LC276985 | 761 | DQ481693.1 | 0.98 |
| 32 | *Cortinarius* sp. 14 |  |  | 2 | 1 |  |  |  | LC276986 | 630 | HQ604715.1 | 0.98 |
| 33 | *Cortinarius* sp. 15 |  |  | 2 |  |  | 2 |  | LC276987 | 618 | KP406540.1 | 0.99 |
| 34 | *Cortinarius* sp. 16 |  |  |  | 1 | 1 |  |  | LC276988 | 561 | KP406537.1 | 0.99 |
| 35 | *Cortinarius* sp. 17 |  | 1 |  |  |  |  |  | LC276989 | 816 | KF617829.1 | 0.97 |
| 36 | *Cortinarius* sp. 18 |  |  | 1 | 1 |  | 1 |  | LC276990 | 742 | AY669680.1 | 0.99 |
| 37 | *Cortinarius* sp. 19 | 1 |  | 1 |  | 1 |  |  | LC276991 | 510 | JX436887.1 | 1 |
| 38 | *Cortinarius* sp. 20 |  |  | 1 | 1 |  |  |  | LC276992 | 361 | EU668910.1 | 0.96 |
| 39 | *Cortinarius* sp. 21 |  |  |  |  | 1 |  |  | LC276993 | 699 | HQ604685.1 | 0.97 |
| 40 | *Cortinarius* sp. 22 |  |  | 1 |  |  |  |  | LC276994 | 541 | GQ159884.1 | 0.99 |
| 41 | *Cortinarius* sp. 23 |  |  |  |  | 1 | 1 |  | LC276995 | 428 | DQ097877.1 | 0.99 |
| 42 | *Cortinarius* sp. 24 |  |  |  |  |  | 1 |  | LC276996 | 659 | EU259285.1 | 0.99 |
| 43 | *Cortinarius* sp. 25 |  |  |  |  | 1 |  |  | LC276997 | 533 | KP406537.1 | 0.98 |
| 44 | *Cortinarius* sp. 26 |  |  |  | 1 |  | 1 |  | LC276998 | 539 | FJ553180.1 | 0.99 |
| 45 | *Cortinarius* sp. 27 |  |  |  |  |  |  | 1 | LC276999 | 482 | JN197989.1 | 0.99 |
| 46 | *Cortinarius* sp. 28 |  |  | 1 |  |  |  |  | LC277000 | 501 | EU517048.1 | 0.98 |
| 47 | *Cortinarius* sp. 29 |  |  | 1 |  |  |  |  | LC277001 | 513 | KJ769270.1 | 0.99 |
| 48 | *Cortinarius* sp. 30 |  |  |  | 1 |  |  |  | LC277002 | 379 | JQ749630.1 | 0.99 |
| 49 | *Cortinarius* sp. 31 |  | 1 |  |  |  |  |  | LC277003 | 469 | AB848454.1 | 0.99 |
| 50 | *Cortinarius* sp. 32 |  |  |  |  | 1 |  |  | LC277004 | 328 | KM576354.1 | 0.99 |
| 51 | *Cortinarius* sp. 33 |  |  |  |  | 1 |  |  | LC277005 | 363 | FJ039708.1 | 0.98 |
| 52 | *Cortinarius* sp. 34 |  |  |  |  |  |  | 1 | LC277006 | 564 | GQ159776.1 | 0.99 |
| 53 | *Craterellus* sp. |  |  | 1 |  |  |  |  | LC277007 | 134 | JQ991715.1 | 0.91 |
| 54 | *Elaphomyces* sp. |  |  |  |  |  | 1 |  | LC277008 | 277 | KM576392.1 | 0.98 |
| 55 | *Entoloma* sp. |  | 1 |  |  |  |  |  | LC277009 | 404 | KJ001414.1 | 0.99 |
| 56 | *Hebeloma* sp. 1 |  | 2 | 3 | 4 | 1 | 1 |  | LC277010 | 772 | DQ822807.1 | 0.99 |
| 57 | *Hebeloma* sp. 2 |  | 1 | 2 | 2 | 1 |  |  | LC277011 | 772 | KJ146712.1 | 0.98 |
| 58 | Helotiales sp. 1 |  |  | 1 |  |  | 1 |  | LC277012 | 505 | JX507662.1 | 0.99 |
| 59 | Helotiales sp. 2 |  |  |  |  | 1 |  |  | LC277013 | 467 | KC876217.1 | 0.98 |
| 60 | *Hyaloscyphaceae* sp. |  |  |  | 1 | 1 |  |  | LC277014 | 373 | EF434141.1 | 0.99 |
| 61 | *Hygrophorus* sp. 1 |  | 1 | 1 | 7 |  |  |  | LC277015 | 837 | HM044495.1 | 0.97 |
| 62 | *Hygrophorus* sp. 2 |  |  |  |  | 1 |  |  | LC277016 | 665 | JF899557.1 | 0.99 |
| 63 | *Hygrophorus* sp. 3 | 1 |  |  | 5 |  |  |  | LC277017 | 613 | HM044495.1 | 0.97 |
| 64 | *Hygrophorus* sp. 4 |  |  |  | 3 |  |  |  | LC277018 | 713 | HM044495.1 | 0.99 |
| 65 | *Hygrophorus* sp. 5 |  |  |  | 1 |  |  |  | LC277019 | 123 | HM044513.1 | 0.98 |
| 66 | *Hygrophorus* sp. 6 |  | 1 |  | 1 |  |  |  | LC277020 | 586 | HM044504.1 | 0.99 |
| 67 | *Hypochnicium sp.* |  |  | 1 |  |  |  |  | LC277021 | 373 | KJ140768.1 | 0.99 |
| 68 | *Inocybe* sp. 1 |  | 1 | 1 | 2 |  |  | 2 | LC277022 | 878 | HQ604286.1 | 0.99 |
| 69 | *Inocybe* sp. 2 |  | 1 | 4 | 1 | 1 |  | 1 | LC277023 | 720 | HQ604751.1 | 0.99 |
| 70 | *Inocybe* sp. 3 |  |  | 1 | 5 | 1 |  |  | LC277024 | 637 | AM882796.2 | 0.97 |
| 71 | *Inocybe* sp. 4 |  |  |  | 1 | 2 |  |  | LC277025 | 730 | HQ604367.1 | 0.99 |
| 72 | *Inocybe* sp. 5 |  |  |  |  | 1 | 1 |  | LC277026 | 635 | JN580835.1 | 0.97 |
| 73 | *Inocybe* sp. 6 |  |  | 1 | 1 |  |  |  | LC277027 | 720 | JF899559.1 | 0.97 |
| 74 | *Inocybe* sp. 7 |  |  |  | 1 |  | 1 |  | LC277028 | 664 | KJ399920.1 | 0.99 |
| 75 | *Inocybe* sp. 8 |  |  |  |  | 1 |  | 1 | LC277029 | 677 | AM882711.2 | 0.97 |
| 76 | *Inocybe* sp. 9 |  |  | 1 |  |  |  |  | LC277030 | 710 | HQ604446.1 | 0.97 |
| 77 | *Inocybe* sp. 10 |  | 1 |  |  |  | 1 |  | LC277031 | 527 | HQ604245.1 | 0.99 |
| 78 | *Inocybe* sp. 11 |  |  |  | 1 | 1 |  |  | LC277032 | 642 | AM882796.2 | 0.98 |
| 79 | *Inocybe* sp. 12 |  |  |  | 1 |  |  |  | LC277033 | 588 | HQ604232.1 | 0.98 |
| 80 | *Inocybe* sp. 13 |  |  |  |  | 1 |  |  | LC277034 | 498 | AB848495.1 | 0.99 |
| 81 | *Inocybe* sp. 14 |  | 1 |  |  |  |  |  | LC277035 | 561 | FN550881.1 | 0.93 |
| 82 | *Laccaria* sp. 1 |  |  | 4 | 1 |  |  |  | LC277036 | 781 | JX030275.1 | 0.99 |
| 83 | *Laccaria* sp. 2 |  |  | 1 |  |  | 3 |  | LC277037 | 743 | AY254869.2 | 0.99 |
| 84 | *Laccaria* sp. 3 |  |  | 2 | 1 |  | 1 |  | LC277038 | 732 | JX316668.1 | 0.99 |
| 85 | *Laccaria* sp. 4 |  |  | 2 |  |  | 1 | 1 | LC277039 | 643 | JF273528.1 | 0.99 |
| 86 | *Laccaria* sp. 5 |  |  | 1 |  |  | 1 |  | LC277040 | 684 | GU998223.1 | 0.99 |
| 87 | *Laccaria* sp. 6 |  |  |  | 2 |  |  | 1 | LC277041 | 735 | EF644110.1 | 0.99 |
| 88 | *Lactarius* sp. 1 |  | 1 |  | 1 |  | 16 |  | LC277042 | 769 | EF218784.1 | 0.99 |
| 89 | *Lactarius* sp. 2 |  |  |  | 4 |  |  |  | LC277043 | 737 | HM044509.1 | 0.99 |
| 90 | *Lactarius* sp. 3 |  |  |  |  | 2 | 1 |  | LC277044 | 727 | DQ097869.1 | 0.98 |
| 91 | *Lactarius* sp. 4 |  |  |  | 1 | 2 | 1 |  | LC277045 | 692 | JN197640.1 | 0.97 |
| 92 | *Lactarius* sp. 5 |  |  |  |  | 1 |  |  | LC277046 | 762 | EU057099.2 | 0.98 |
| 93 | *Lactarius* sp. 6 |  |  |  |  | 1 |  |  | LC277047 | 667 | KJ705213.1 | 0.98 |
| 94 | *Lactarius* sp. 7 |  |  |  | 1 | 1 |  |  | LC277048 | 503 | KM069448.1 | 0.98 |
| 95 | *Lactarius* sp. 8 |  |  |  |  |  | 1 |  | LC277049 | 682 | GU373496.1 | 0.98 |
| 96 | *Lactarius* sp. 9 |  |  |  |  | 1 |  |  | LC277050 | 639 | KJ742389.1 | 0.99 |
| 97 | *Leccinum* sp. 1 |  |  |  |  |  | 2 |  | LC277051 | 661 | KC552011.1 | 0.96 |
| 98 | *Leccinum* sp. 2 |  | 1 |  |  |  |  |  | LC277052 | 701 | AF454572.1 | 0.99 |
| 99 | *Leotiomycetes* sp. |  |  |  | 2 |  |  |  | LC277053 | 495 | LC035152.1 | 0.99 |
| 100 | *Meliniomyces* sp. |  |  |  |  |  | 1 |  | LC277054 | 502 | HM044588.1 | 0.98 |
| 101 | *Phialocephala* sp. | 4 | 1 | 1 | 1 |  |  |  | LC277055 | 491 | JQ346953.1 | 0.99 |
| 102 | *Piloderma* sp. 1 |  |  |  | 1 | 3 | 1 | 2 | LC277056 | 743 | JQ711815.1 | 0.99 |
| 103 | *Piloderma* sp. 2 |  | 1 |  |  | 2 |  |  | LC277057 | 764 | DQ469288.1 | 0.96 |
| 104 | *Piloderma* sp. 3 |  | 1 |  |  | 3 |  |  | LC277058 | 777 | FJ236851.1 | 0.98 |
| 105 | *Piloderma* sp. 4 |  |  |  |  |  | 1 |  | LC277059 | 635 | FR852330.1 | 0.92 |
| 106 | *Piloderma* sp. 5 |  | 1 |  |  | 1 |  |  | LC277060 | 618 | EF434022.1 | 0.99 |
| 107 | *Piloderma* sp. 6 |  |  |  |  | 1 |  |  | LC277061 | 695 | AB848555.1 | 0.99 |
| 108 | *Piloderma* sp. 7 |  |  |  |  |  |  | 1 | LC277062 | 536 | AJ534902.1 | 0.99 |
| 109 | *Pseudotomentella* sp. |  |  | 1 |  |  |  |  | LC277063 | 513 | GU134527.1 | 0.89 |
| 110 | *Ramariopsis* sp. |  |  |  |  | 1 |  |  | LC277064 | 316 | KJ146701.1 | 0.98 |
| 111 | *Russula* sp. 1 | 1 |  | 2 | 9 | 1 | 1 | 3 | LC277065 | 712 | GU452511.1 | 0.99 |
| 112 | *Russula* sp. 2 |  | 1 | 1 |  | 1 |  |  | LC277066 | 500 | KP226191.1 | 0.99 |
| 113 | *Russula* sp. 3 | 1 |  |  | 3 |  |  |  | LC277067 | 836 | KM402893.1 | 0.99 |
| 114 | *Russula* sp. 4 |  |  | 3 |  | 1 |  |  | LC277068 | 744 | FJ845431.1 | 0.99 |
| 115 | *Russula* sp. 5 |  |  |  |  | 4 |  |  | LC277069 | 779 | HQ604850.1 | 0.97 |
| 116 | *Russula* sp. 6 |  |  |  |  | 1 |  |  | LC277070 | 707 | EU057119.2 | 0.99 |
| 117 | *Russula* sp. 7 |  |  | 2 | 1 |  |  |  | LC277071 | 625 | FR852095.1 | 0.99 |
| 118 | *Russula* sp. 8 |  | 1 |  |  |  |  |  | LC277072 | 680 | AJ534937.1 | 0.99 |
| 119 | *Russula* sp. 9 | 1 |  |  |  |  |  |  | LC277073 | 119 | KF810136.1 | 0.97 |
| 120 | *Russula* sp. 10 |  |  | 1 |  |  |  |  | LC277074 | 134 | JQ991792.1 | 0.99 |
| 121 | *Russula* sp. 11 |  | 1 |  |  |  |  |  | LC277075 | 479 | KP226186.1 | 0.99 |
| 122 | *Russula* sp. 12 |  |  |  |  | 1 |  |  | LC277076 | 493 | AB848586.1 | 1 |
| 123 | *Sebacina* sp. 1 | 1 |  | 3 | 2 | 1 |  | 1 | LC277077 | 700 | JQ711843.1 | 0.99 |
| 124 | *Sebacina* sp. 2 | 1 |  | 1 |  |  |  |  | LC277078 | 281 | AB831800.1 | 0.99 |
| 125 | *Sebacina* sp. 3 |  | 2 |  |  |  |  |  | LC277079 | 157 | JQ666715.1 | 0.97 |
| 126 | *Sebacina* sp. 4 |  |  | 1 |  |  |  |  | LC277080 | 134 | JX844775.1 | 0.97 |
| 127 | *Sebacina* sp. 5 |  | 1 |  |  |  |  |  | LC277081 | 634 | KM403000.1 | 0.98 |
| 128 | *Sebacina* sp. 6 |  | 1 |  |  |  |  |  | LC277082 | 578 | HQ154331.1 | 0.98 |
| 129 | *Sebacina* sp. 7 |  |  |  |  |  | 1 | 1 | LC277083 | 534 | AB807999.1 | 0.98 |
| 130 | *Sebacina* sp. 8 |  |  | 1 |  |  |  |  | LC277084 | 519 | AB848613.1 | 0.99 |
| 131 | *Sebacina* sp. 9 |  |  | 2 |  |  |  |  | LC277085 | 515 | JX844772.1 | 1 |
| 132 | *Sebacina* sp. 10 | 1 |  |  |  |  |  |  | LC277086 | 331 | JQ420983.1 | 0.96 |
| 133 | *Suillus* sp. 1 |  |  |  | 6 |  |  | 1 | LC277087 | 729 | AF166505.2 | 0.99 |
| 134 | *Suillus* sp. 2 |  |  |  | 4 |  |  | 1 | LC277088 | 733 | GU181857.1 | 0.98 |
| 135 | *Suillus* sp. 3 |  |  |  | 2 |  |  |  | LC277089 | 622 | JN021099.1 | 0.99 |
| 136 | Thelephoraceae sp. 1 |  | 1 | 6 | 3 | 2 | 3 | 1 | LC277090 | 830 | AJ893340.1 | 0.97 |
| 137 | Thelephoraceae sp. 2 |  |  | 5 | 1 |  |  | 1 | LC277091 | 710 | JX030226.1 | 0.99 |
| 138 | Thelephoraceae sp. 3 |  |  | 3 |  | 2 | 1 |  | LC277092 | 847 | FN669269.1 | 0.99 |
| 139 | Thelephoraceae sp. 4 |  |  | 1 | 2 | 2 | 1 |  | LC277093 | 814 | AJ893343.1 | 0.99 |
| 140 | Thelephoraceae sp. 5 |  |  | 1 |  | 1 |  | 1 | LC277094 | 703 | FJ816773.1 | 0.99 |
| 141 | Thelephoraceae sp. 6 | 1 |  |  |  |  |  | 2 | LC277095 | 781 | HE979096.1 | 0.99 |
| 142 | Thelephoraceae sp. 7 |  |  |  |  | 4 | 1 |  | LC277096 | 726 | EF218840.1 | 0.99 |
| 143 | Thelephoraceae sp. 8 |  | 1 | 4 |  |  |  |  | LC277097 | 753 | DQ482000.1 | 0.97 |
| 144 | Thelephoraceae sp. 9 |  |  | 1 | 1 | 1 |  |  | LC277098 | 846 | EF218819.1 | 0.98 |
| 145 | Thelephoraceae sp. 10 |  |  |  | 1 | 2 |  |  | LC277099 | 814 | FN669278.1 | 0.99 |
| 146 | Thelephoraceae sp. 11 |  |  | 1 | 1 |  |  |  | LC277100 | 804 | KM403055.1 | 0.96 |
| 147 | Thelephoraceae sp. 12 |  | 1 | 1 | 4 |  |  |  | LC277101 | 757 | FN669271.1 | 0.96 |
| 148 | Thelephoraceae sp. 13 |  |  |  |  | 2 |  |  | LC277102 | 756 | FN669280.1 | 0.98 |
| 149 | Thelephoraceae sp. 14 |  |  | 2 |  |  |  |  | LC277103 | 710 | KM402978.1 | 0.99 |
| 150 | Thelephoraceae sp. 15 |  |  | 1 |  | 2 |  |  | LC277104 | 766 | KJ769317.1 | 0.94 |
| 151 | Thelephoraceae sp. 16 |  |  | 1 |  |  | 1 |  | LC277105 | 721 | JQ393128.1 | 0.97 |
| 152 | Thelephoraceae sp. 17 |  |  | 1 |  | 2 |  |  | LC277106 | 706 | KM402890.1 | 0.98 |
| 153 | Thelephoraceae sp. 18 |  | 1 | 1 | 1 | 1 |  |  | LC277107 | 657 | EF218833.1 | 0.99 |
| 154 | Thelephoraceae sp. 19 |  |  | 2 |  |  |  |  | LC277108 | 610 | EF644148.1 | 0.99 |
| 155 | Thelephoraceae sp. 20 |  | 1 | 1 |  |  |  |  | LC277109 | 747 | FN669278.1 | 0.96 |
| 156 | Thelephoraceae sp. 21 |  |  | 1 |  | 1 |  |  | LC277110 | 389 | AF272931.1 | 0.97 |
| 157 | Thelephoraceae sp. 22 |  |  |  |  |  |  | 1 | LC277111 | 780 | JQ890239.1 | 0.99 |
| 158 | Thelephoraceae sp. 23 |  | 1 | 1 |  | 1 |  |  | LC277112 | 669 | JQ890244.1 | 0.98 |
| 159 | Thelephoraceae sp. 24 |  |  | 1 |  |  |  |  | LC277113 | 184 | AB669658.1 | 0.98 |
| 160 | Thelephoraceae sp. 25 |  |  |  |  | 1 |  |  | LC277114 | 544 | JQ711813.1 | 0.96 |
| 161 | Thelephoraceae sp. 26 |  |  |  | 1 | 1 |  |  | LC277115 | 382 | AB848675.1 | 0.99 |
| 162 | Thelephoraceae sp. 27 |  |  |  | 1 |  |  |  | LC277116 | 901 | FN669257.1 | 0.98 |
| 163 | Thelephoraceae sp. 28 |  |  |  |  |  | 1 |  | LC277117 | 675 | KF514693.1 | 0.98 |
| 164 | Thelephoraceae sp. 29 |  |  |  |  | 1 |  |  | LC277118 | 696 | JX030242.1 | 1 |
| 165 | Thelephoraceae sp. 30 |  |  | 1 |  |  |  |  | LC277119 | 413 | KM981765.1 | 0.99 |
| 166 | Thelephoraceae sp. 31 |  |  |  |  | 1 |  |  | LC277120 | 316 | AB848665.1 | 1 |
| 167 | Thelephoraceae sp. 32 |  | 1 |  |  |  |  |  | LC277121 | 274 | GU220622.1 | 0.99 |
| 168 | Thelephoraceae sp. 33 |  |  | 1 |  |  |  |  | LC277122 | 408 | EU526858.1 | 0.99 |
| 169 | Thelephoraceae sp. 34 |  |  | 1 |  |  |  |  | LC277123 | 438 | GU969269.1 | 0.99 |
| 170 | Thelephoraceae sp. 35 |  |  |  |  |  |  | 1 | LC277124 | 493 | HE814198.1 | 1 |
| 171 | Thelephoraceae sp. 36 |  |  |  | 1 |  |  |  | LC277125 | 539 | GU134522.1 | 0.95 |
| 172 | Thelephoraceae sp. 37 |  |  |  |  |  | 1 |  | LC277126 | 628 | FN669269.1 | 1 |
| 173 | *Tomentellopsis* sp. |  |  |  | 1 |  |  |  | LC277127 | 503 | KP403093.1 | 0.99 |
| 174 | *Tricholoma* sp. 1 |  |  |  |  |  | 1 |  | LC277128 | 590 | AB036899.1 | 0.99 |
| 175 | *Tricholoma* sp. 2 |  |  |  |  | 1 |  |  | LC277129 | 507 | KP406555.1 | 0.99 |
| 176 | *Wilcoxina* sp. 1 | 6 |  |  | 3 |  |  |  | LC277130 | 552 | FM992988.1 | 0.99 |
| 177 | *Wilcoxina* sp. 2 | 5 |  |  |  |  |  |  | LC277131 | 550 | EU668262.1 | 0.99 |
| 178 | *Wilcoxina* sp. 3 | 1 |  |  |  |  |  |  | LC277132 | 537 | FM992988.1 | 0.97 |
| 179 | *Tuber* sp. |  |  |  |  |  |  | 1 | LC277133 | 605 | JQ712002.1 | 0.99 |
| 180 | *Xerocomus* sp. |  |  |  |  |  |  | 1 | LC277134 | 568 | AB848704.1 | 0.99 |
|  | Fungal species richness | 18 | 42 | 75 | 69 | 73 | 44 | 34 |  |  |  |  |
| 1. Total number of plant individuals colonized by each fungal species is shown. | | | | | | | | | | | | |
| 2. Abbreviations of Pyroleae species: *Os*, *Orthilia secunda*; *Pa*, *Pyrola alpina*; *Pi*, *Pyrola incarnata*. | | | | | | | | | | | | |
| 3. Abbreviations of Ectomycorrhizal trees: *L*, *Larix*; *A*, *Abies*; *B*, *Betula*; O, Other trees. | | | | | | | | | | | | |
