# Supplementary material for: Root-associated fungal communities in three Pyroleae species and their mycobiont sharing with surrounding trees in subalpine coniferous forests on Mount Fuji, Japan
Source: Mycorrhiza. 2017 Jul 13;27(8):733–45. doi: 10.1007/s00572-017-0788-6 (PMC5645451; doi:10.1007/s00572-017-0788-6)
Supplement: Supplementary file 5 — (DOCX 22 kb) [file 572_2017_788_MOESM5_ESM.docx]

| **Table S3** Fungal lineages confirmed in roots of Pyroleae and ectomycorrhizal trees in subalpine forests on Mount Fuji, Japan. | | | | |
| --- | --- | --- | --- | --- |
| Host plants | Fungal lineages (ID No.) | | Division | Occurrence* |
| *O. secunda* | *Amphinema* | 2 | Basidiomycota | 2 |
| *O. secunda* | Aphyllophorales | 3 | Basidiomycota | 1 |
| *O. secunda* | *Boletus* | 4 | Basidiomycota | 1 |
| *O. secunda* | *Cortinarius* | 10 | Basidiomycota | 3 |
| *O. secunda* | *Hygrophorus* | 18 | Basidiomycota | 1 |
| *O. secunda* | *Phialocephala* | 26 | Ascomycota | 4 |
| *O. secunda* | *Russula* | 29 | Basidiomycota | 3 |
| *O. secunda* | *Sebacina* | 30 | Basidiomycota | 3 |
| *O. secunda* | Thelephoraceae | 32 | Basidiomycota | 1 |
| *O. secunda* | *Wilcoxina* | 35 | Ascomycota | 12 |
| *P. alpina* | *Amphinema* | 2 | Basidiomycota | 8 |
| *P. alpina* | *Boletus* | 4 | Basidiomycota | 1 |
| *P. alpina* | *Cantharellales* | 5 | Basidiomycota | 1 |
| *P. alpina* | *Cenococcum* | 6 | Ascomycota | 1 |
| *P. alpina* | *Clavulina* | 8 | Basidiomycota | 1 |
| *P. alpina* | *Clitocybe* | 9 | Basidiomycota | 1 |
| *P. alpina* | *Cortinarius* | 10 | Basidiomycota | 14 |
| *P. alpina* | *Entoloma* | 13 | Basidiomycota | 1 |
| *P. alpina* | *Hebeloma* | 14 | Basidiomycota | 3 |
| *P. alpina* | *Hygrophorus* | 18 | Basidiomycota | 2 |
| *P. alpina* | *Inocybe* | 20 | Basidiomycota | 4 |
| *P. alpina* | *Lactarius* | 22 | Basidiomycota | 1 |
| *P. alpina* | *Leccinum* | 23 | Basidiomycota | 1 |
| *P. alpina* | *Phialocephala* | 26 | Ascomycota | 1 |
| *P. alpina* | *Piloderma* | 27 | Basidiomycota | 3 |
| *P. alpina* | *Russula* | 29 | Basidiomycota | 3 |
| *P. alpina* | *Sebacina* | 30 | Basidiomycota | 4 |
| *P. alpina* | Thelephoraceae | 32 | Basidiomycota | 8 |
| *P. incarnata* | *Amphinema* | 2 | Basidiomycota | 9 |
| *P. incarnata* | Aphyllophorales | 3 | Basidiomycota | 1 |
| *P. incarnata* | *Cenococcum* | 6 | Ascomycota | 1 |
| *P. incarnata* | *Cortinarius* | 10 | Basidiomycota | 55 |
| *P. incarnata* | *Craterellus* | 11 | Basidiomycota | 1 |
| *P. incarnata* | *Hebeloma* | 14 | Basidiomycota | 5 |
| *P. incarnata* | Helotiales | 15 | Ascomycota | 1 |
| *P. incarnata* | *Hygrophorus* | 18 | Basidiomycota | 1 |
| *P. incarnata* | *Hypochnicium* | 19 | Basidiomycota | 1 |
| *P. incarnata* | *Inocybe* | 20 | Basidiomycota | 9 |
| *P. incarnata* | *Laccaria* | 21 | Basidiomycota | 10 |
| *P. incarnata* | *Phialocephala* | 26 | Ascomycota | 1 |
| *P. incarnata* | *Russula* | 29 | Basidiomycota | 9 |
| *P. incarnata* | *Sebacina* | 30 | Basidiomycota | 8 |
| *P. incarnata* | Thelephoraceae | 32 | Basidiomycota | 38 |
| *Abies* | *Amanita* | 1 | Basidiomycota | 2 |
| *Abies* | *Boletus* | 4 | Basidiomycota | 5 |
| *Abies* | *Cenococcum* | 6 | Ascomycota | 28 |
| *Abies* | *Clavulina* | 8 | Basidiomycota | 1 |
| *Abies* | *Cortinarius* | 10 | Basidiomycota | 28 |
| *Abies* | *Hebeloma* | 14 | Basidiomycota | 2 |
| *Abies* | Helotiales | 15 | Ascomycota | 1 |
| *Abies* | Hyaloscyphaceae | 16 | Ascomycota | 1 |
| *Abies* | *Hygrocybe* | 17 | Basidiomycota | 1 |
| *Abies* | *Hygrophorus* | 18 | Basidiomycota | 1 |
| *Abies* | *Inocybe* | 20 | Basidiomycota | 10 |
| *Abies* | *Lactarius* | 22 | Basidiomycota | 8 |
| *Abies* | *Piloderma* | 27 | Basidiomycota | 10 |
| *Abies* | *Ramariopsis* | 28 | Basidiomycota | 1 |
| *Abies* | *Russula* | 29 | Basidiomycota | 10 |
| *Abies* | *Sebacina* | 30 | Basidiomycota | 2 |
| *Abies* | *Suillus* | 31 | Basidiomycota | 2 |
| *Abies* | Thelephoraceae | 32 | Basidiomycota | 24 |
| *Abies* | *Tricholoma* | 33 | Basidiomycota | 1 |
| *Betula* | *Cenococcum* | 6 | Ascomycota | 6 |
| *Betula* | *Clavulina* | 8 | Basidiomycota | 1 |
| *Betula* | *Cortinarius* | 10 | Basidiomycota | 21 |
| *Betula* | *Elaphomyces* | 12 | Ascomycota | 1 |
| *Betula* | *Hebeloma* | 14 | Basidiomycota | 1 |
| *Betula* | Helotiales | 15 | Ascomycota | 1 |
| *Betula* | *Inocybe* | 20 | Basidiomycota | 3 |
| *Betula* | *Laccaria* | 21 | Basidiomycota | 6 |
| *Betula* | *Lactarius* | 22 | Basidiomycota | 18 |
| *Betula* | *Leccinum* | 23 | Basidiomycota | 1 |
| *Betula* | *Meliniomyces* | 25 | Ascomycota | 1 |
| *Betula* | *Piloderma* | 27 | Basidiomycota | 2 |
| *Betula* | *Sebacina* | 30 | Basidiomycota | 1 |
| *Betula* | Thelephoraceae | 32 | Basidiomycota | 7 |
| *Betula* | *Tricholoma* | 33 | Basidiomycota | 1 |
| *Larix* | *Amphinema* | 2 | Basidiomycota | 1 |
| *Larix* | *Boletus* | 4 | Basidiomycota | 3 |
| *Larix* | *Cenococcum* | 6 | Ascomycota | 14 |
| *Larix* | *Chroogomphus* | 7 | Basidiomycota | 1 |
| *Larix* | *Clavulina* | 8 | Basidiomycota | 1 |
| *Larix* | *Cortinarius* | 10 | Basidiomycota | 52 |
| *Larix* | *Hebeloma* | 14 | Basidiomycota | 6 |
| *Larix* | Hyaloscyphaceae | 16 | Ascomycota | 1 |
| *Larix* | *Hygrophorus* | 18 | Basidiomycota | 18 |
| *Larix* | *Inocybe* | 20 | Basidiomycota | 13 |
| *Larix* | *Laccaria* | 21 | Basidiomycota | 4 |
| *Larix* | *Lactarius* | 22 | Basidiomycota | 7 |
| *Larix* | Leotiomycetes | 24 | Ascomycota | 2 |
| *Larix* | *Phialocephala* | 26 | Ascomycota | 1 |
| *Larix* | *Piloderma* | 27 | Basidiomycota | 1 |
| *Larix* | *Russula* | 29 | Basidiomycota | 16 |
| *Larix* | *Sebacina* | 30 | Basidiomycota | 3 |
| *Larix* | *Suillus* | 31 | Basidiomycota | 12 |
| *Larix* | Thelephoraceae | 32 | Basidiomycota | 18 |
| *Larix* | *Tuber* | 34 | Ascomycota | 1 |
| *Larix* | *Wilcoxina* | 35 | Ascomycota | 3 |
| *Larix* | *Xerocomus* | 36 | Basidiomycota | 1 |
| * The occurrence of fungal species (the number of soil blocks containing that species) were pooled for each lineage/host plants. | | | | |
| Fungal ID numbers corresponds with Fig.S2. | | | | |
